# Trend in cancer incidence and mortality among children and adolescents in Mato Grosso, 2001–2018

**DOI:** 10.1590/S2237-96222025v34e20250146.en

**Published:** 2025-10-03

**Authors:** Mariana Rosa Soares, Pablo Cardozo Roccon, Wanderlei Antonio Pignati, Noemi Dreyer Galvão, Paulo César Fernandes de Souza, Amanda Cristina de Souza Andrade

**Affiliations:** 1 Universidade Federal de Mato Grosso, Instituto de Saúde Coletiva, Cuiabá, MT, Brazil; 2 Secretaria de Saúde do Estado de Mato Grosso, Cuiabá, MT, Brazil; 3 Instituto René Rachou, Fundação Oswaldo Cruz, Belo Horizonte, MG, Brazil

**Keywords:** Incidence, Neoplasms, Child, Adolescent, Time Series Studies, Incidencia, Neoplasias, Niño, Adolescente, Estudios de Series Temporales

## Abstract

**Objective:**

To analyze the temporal trend of cancer incidence and mortality among children and adolescents residing in Mato Grosso during the period 2001–2018.

**Methods:**

This was a time series study of cancer incidence and mortality rates among children and adolescents aged 0–19 years residing in Mato Grosso. Joinpoint regression was used to estimate the annual percentage changes (APC) and 95% confidence intervals (95%CI).

**Results:**

A total of 1,915 new cases and 796 deaths were recorded, with leukemias being the most common (30.23% of cases and 34.92% of deaths) for the 15–19 age group (28.25% of cases and 30.78% of deaths), among individuals of Brown race/skin color (45.17% of cases and 50.75% of deaths), and males (53.89% of cases and 56.53% of deaths). Incidence rates showed a decreasing trend for leukemias between 2001 and 2010 (APC -5.5%; 95%CI -8.3; -2.6) and soft tissue sarcomas (APC -7.3%; 95%CI -10.6; -3.8) during the period 2001-2018, as well as for all causes between 2001 and 2016 (APC -1.1%; 95%CI -2.0; -0.2). Mortality rates remained stable throughout the period and for all types of cancer.

**Conclusion:**

Although stable, the rates in Mato Grosso are high, which calls for strategies to promote access to early diagnosis and timely, appropriate treatment, such as expanding the network of specialized services, improving the quality of these services, and updating the data.

## Resumen

**Objetivo:**

Analizar la tendencia temporal de la incidencia y mortalidad por cáncer en niños y adolescentes residentes en Mato Grosso, durante el período de 2001 a 2018.

**Métodos:**

Estudio de series temporales de las tasas de incidencia y mortalidad por cáncer en niños y adolescentes de 0 a 19 años residentes en el estado de Mato Grosso, Brasil. Para estimar las variaciones porcentuales anuales (APC) y los intervalos de confianza del 95% (IC95%), se utilizó la regresión joinpoint.

**Resultados:**

Se registraron 1.915 casos nuevos y 796 muertes. Los tipos más incidentes fueron las leucemias (30,23% de los casos y 34,92% de los óbitos), en el grupo de edad de 15 a 19 años (28,25% de los casos y 30,78% de las muertes), en personas de raza/color de piel parda (45,17% de los casos y 50,75% de las muertes) y en el sexo masculino (53,89% de los casos y 56,53% de las muertes). Las tasas de incidencia presentaron tendencia descendente para las leucemias entre 2001 y 2010 (APC -5,5%; IC95% -8,3; -2,6) y para los sarcomas de partes blandas (APC -7,3%; IC95% -10,6; -3,8) durante 2001-2018, así como para todas las causas entre 2001 y 2016 (APC -1,1%; IC95% -2,0; -0,2). Las tasas de mortalidad se mantuvieron estables en todo el período y para todos los tipos de cáncer.

**Conclusione:**

s: A pesar de la estabilidad observada, las tasas en Mato Grosso siguen siendo elevadas, lo que requiere estrategias que promuevan el acceso al diagnóstico precoz y al tratamiento adecuado en el tiempo oportuno, como la ampliación de la red de servicios especializados, la mejora en la calidad de dichos servicios y la actualización de los datos disponibles.

**Ethical aspects**

This research respected ethical principles, having obtained the following approval data:

Research Ethics Committee: Universidade Federal de Mato Grosso

Opinion number: 4,858,521

Approval date: 20/7/2021

Certificate of Submission for Ethical Appraisal: 48121421.0.0000.8124

Informed Consent Form: Exempt.

## Introduction

Childhood and adolescent cancer is a chronic disease that affects children and adolescents aged 0–19 years (1). In this age group, the most frequent types of tumors are leukemias, lymphomas, central nervous system tumors, sarcomas, and bone tumors (2).

In the global scenario, there is an increase in cancer incidence and mortality among children and adolescents, with more than 400,000 new cases and 73,000 deaths. Of this total, 80.0% occur in countries with low Human Development Index (HDI) (3). In Brazil, an estimated 4,230 cases are expected for males and 3,700 for females for the 2023–2025 three-year period (1). For the same period, the highest crude incidence rates were estimated in the South, at 152.26 per 1 million inhabitants, followed by the Southeast (144.88 per 1,000,000 inhabitants) and the Central-West (136.21 per 1,000,000 inhabitants), where Mato Grosso is located, a state with a crude rate of 115.41 per 1,000,000 inhabitants (1). In Mato Grosso, among the most incident types between 2001 and 2017, leukemias and lymphomas stood out, with age-adjusted incidence rates of 29.8 and 13.9 cases per 1,000,000 inhabitants.

Unlike cancers in adults, in this age group, tumors have their own clinical characteristics, due to their etiological origin, clinical behavior, and associated factors (5). Regarding clinical behavior and prognosis, improvements in cure rates and reductions in mortality have been observed in developed countries. This results from better access to early diagnosis—based on signs and symptoms that may initially be very nonspecific—and timely treatment, which corroborates the stability of mortality rates in these locations (1,4,5). Regarding associated factors, notable ones include genetic, hereditary, and immunological predispositions; environmental exposure to genotoxic agents; ionizing radiation; electromagnetic fields; tobacco use; alcohol consumption during the preconception phase (6,9); and parental exposure to carcinogenic agents in both home and occupational environments (7,9).

Considering the effects of parental environmental and occupational exposure to genotoxic agents on the occurrence of cancer among children and adolescents (6–9), analyzing the state of Mato Grosso is of great scientific and public health relevance. Mato Grosso is a major Brazilian producer of agricultural commodities, operating within a chemically dependent agribusiness production chain and annually consuming large quantities of pesticides (9,10). 

The literature has highlighted cases of cancer in both adults and children resulting from environmental exposure due to living within 90 to 300 meters of crop fields in high-agricultural-production municipalities in the state, as well as from parental occupational exposure during the preconception or gestational periods (9). In this sense, children and adolescents who live in or near cultivated areas may be exposed to the genotoxic effects of pesticides due to exposure caused by drift and contact with contaminated water and soil (10). 

This article aimed to analyze the temporal trends in incidence and mortality of the five most common types of cancer among children and adolescents residing in Mato Grosso between 2001 and 2018.

## Methods

### Study design and setting

This was a time-series study using data from the 141 municipalities of Mato Grosso. Mato Grosso is located in the central region of Brazil and has a territorial area of 903,208.362 km^2^, an estimated population of 3,658,649 inhabitants, a Human Development Index (HDI) of 0.736, and a population density of 4.05 inhabitants/km^2^ (11).

### Participants and variables

The study comprised data from children and adolescents aged 0–19 years, born and residing in the 141 municipalities of Mato Grosso, who were diagnosed with cancer, as well as records of cancer-related deaths in the same age group occurring between 2001 and 2018. The time cut was due to the availability and updating of the population-based cancer registries of the National Cancer Institute (*Instituto Nacional do Câncer*, INCA).

Sociodemographic variables included: age group in years (<1, 0–4, 5–9, 10–14 and 15–19), gender (female, male), race/skin color (Asian, White, Indigenous, Brown, Black) and type of cancer according to diagnosis group according to the International Classification of Childhood Cancer (ICCC) (12).

### Data sources and measurement

Incidence data were extracted from the Population-Based Cancer Registries (*Registros de Câncer de Base Populacional*, RCBP), available on BasepopWeb (13). Death data were taken from the National Mortality Information System (*Sistema de Informações sobre Mortalidade*, SIM) (14), provided by the State Health Department of Mato Grosso. Intercensal population estimates were obtained from the Informatics department of the Brazilian National Health System Information Technology Department (*Departamento de Informática do Sistema Único de Saúde*, Datasus), data in the public domain (15). Incidence and mortality rates (per 1,000,000 inhabitants aged 0–19 years) were calculated for each year between 2001 and 2018 and for the five most common diagnostic groups. Specific crude rates were obtained for each age group using five-year intervals and standardized by age, by the direct method, considering the world standard population (16). 

### Statistical methods

For the trend analysis, joinpoint regression was used, considering the calendar year as the independent variable. The annual percent change (APC) and the average annual percent change (AAPC) were calculated—the latter being the weighted geometric mean of the different annual percent changes, with weights equal to the segment length for each time interval (17). The respective 95% confidence intervals (95%CI) were estimated. Due to the presence of zero values, joinpoint regression for the mortality rate of soft tissue sarcomas was not performed. A significance level of 0.05 was considered.

The rates and graphs were calculated and generated using the R statistical software, version 3.3.0. The analyses were performed using the Joinpoint Regression Program, version 8.3.6.1 (Statistical Research and Applications Branch, National Cancer Institute, Bethesda, United States).

## Results

Between 2001 and 2018, there were 1,915 new cases and 796 deaths from childhood and adolescent cancer in Mato Grosso. The highest proportions were observed in the 15–19 age group (28.25% of cases and 30.78% of deaths) and among males (53.89% of cases and 56.53% of deaths). Brown race/skin color accounted for 45.17% of incident cases and 50.75% of deaths. The most common cancer cases were leukemias (30.23%), central nervous system tumors (15.93%), lymphomas (13.99%), bone tumors (7.89%), and soft tissue sarcomas (5.27%). Among the cancer types of deaths, leukemias (34.92%), tumors of the central nervous system (22.74%), lymphomas (8.54%), malignant bone tumors (7.89%), and soft tissue sarcomas and other extraosseous sarcomas (5.28%) stood out (Table 1).

**Table 1:** Absolute and relative frequencies of new cases and deaths from cancer among children and adolescents. Mato Grosso, 2001–2018 (n=2,771)

Variables	New cases	Deaths
	n (%)	n (%)
**Age group** (years)		
<1	92 (4.80)	29 (3.64)
1-4	480 (25.07)	189 (23.74)
5-9	403 (21.04)	161 (20.23)
10-14	399 (20.84)	172 (21.61)
15-19	541 (28.25)	245 (30.78)
Sex		
Female	883 (46.11)	346 (43.47)
Male	1,032 (53.89)	450 (56.53)
**Race/skin color**		
Asian	27 (1.41)	2 (0.25)
White	616 (32.17)	326 (40.95)
Indigenous	21 (1.10)	13 (1.63)
Black	78 (4.07)	28 (3.52)
Brown	865 (45.17)	404 (50.75)
No information	308 (16.08)	23 (2.89)
Cause		
Leukemias	579 (30.23)	278 (34.92)
Lymphomas and reticuloendothelial neoplasms	268 (13.99)	68 (8.54)
Central nervous system tumors and intracranial and intraspinal neoplasms	305 (15.93)	181 (22.74)
Retinoblastoma	25 (1.31)	6 (0.75)
Kidney tumors	99 (5.17)	14 (1.76)
Liver tumors	20 (1.04)	14 (1.76)
Malignant bone tumors	151 (7.89)	57 (7.16)
Soft tissue sarcomas and other extraosseous sarcomas	101 (5.27)	42 (5.28)
Germ cell tumors, trophoblastic tumors, and gonadal neoplasms	92 (4.80)	20 (2.51)
Other malignant epithelial neoplasms and malignant melanomas	90 (4.70)	31 (3.89)
Other unspecified malignant neoplasms	103 (5.38)	35 (4.40)
Tumors of the sympathetic nervous system	82 (4.28)	50 (6.28)

**Figure 1:**
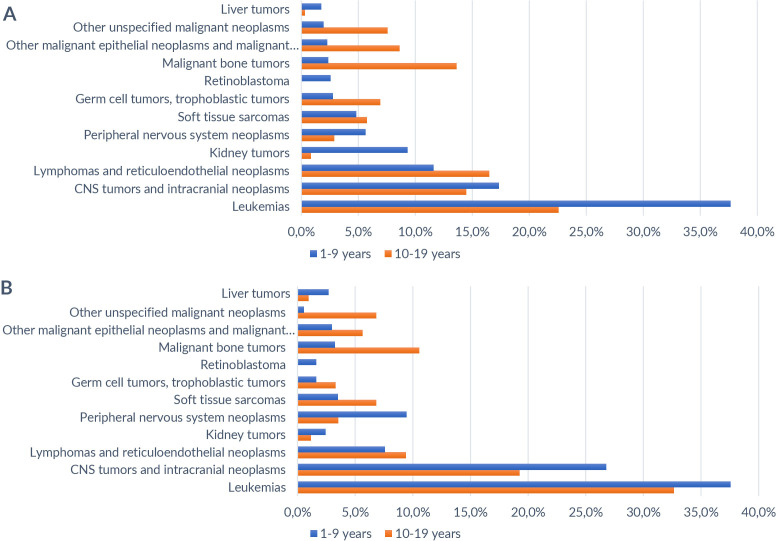
Proportion (A) of new cancer cases and deaths (B) among children and adolescents. Mato Grosso, 2001–2018 (n=2,771)

The highest incidence and mortality rates were observed among children aged 0–9 years compared to adolescents aged 10–19 years. For the diagnostic groups, leukemia accounted for 37.0% of both incidence and mortality, while central nervous system tumors showed an incidence of 17.3% and a mortality of 26.8% among children aged 0–9 years. For the 10–19 years age group, the highest proportions of new cases and deaths were observed in lymphomas (16.5% and 9.4%) and malignant bone tumors (13.6% and 10.6%) (Figure 1).

**Figure 2:**
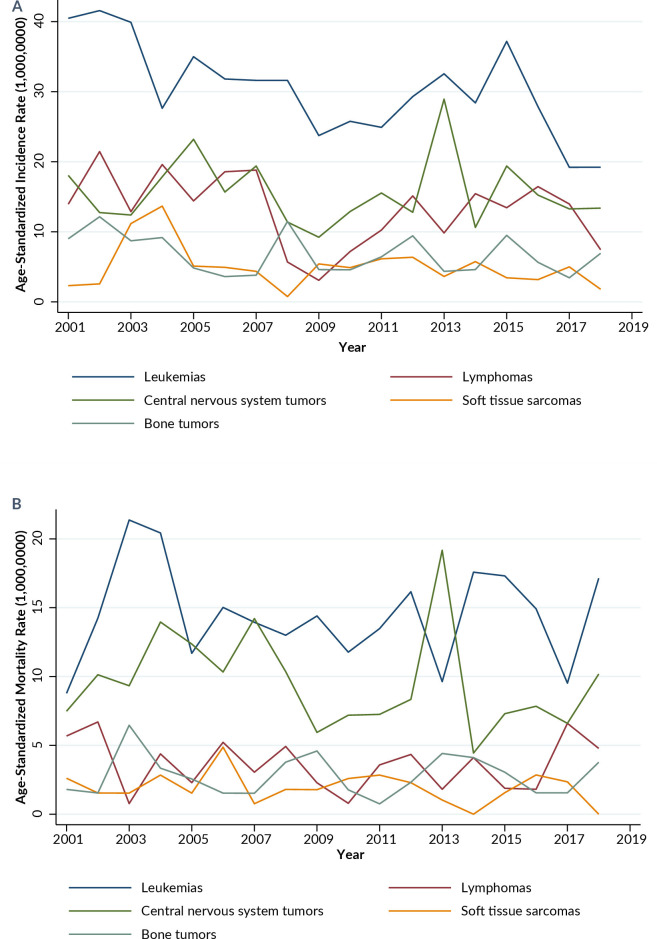
Age-standardized incidence (A) and mortality (B) rates of the five most common types of cancer (per 1,000,000 children and adolescents). Mato Grosso, 2001–2018

The age-standardized incidence rates for the five most frequent types of cancer were observed (Figure 2). For leukemias, the incidence rate was 40.4 cases per 1,000,000 inhabitants in 2001, while the mortality rate was 21.3 per 1,000,000 inhabitants in 2003. For lymphomas, the highest incidence was recorded in 2002, with 21.4 cases per 1,000,000 inhabitants, and the highest mortality occurred in 2016, with 16.4 per 1,000,000 inhabitants. Among central nervous system tumors, the highest incidence was in 2013, with 28.9 cases per 1,000,000 inhabitants, and the highest mortality also occurred in 2013, with 19.2 per 1,000,000 inhabitants. Soft tissue sarcomas had an incidence of 6.4 per 1,000,000 inhabitants and a mortality of 4.9 per 1,000,000 inhabitants in 2006. The highest incidence of bone tumors occurred in 2008, with 11.5 cases per 1,000,000 inhabitants, and the mortality was 6.5 per 1,000,000 inhabitants in 2003.

The trend of the age-standardized incidence rate of leukemias showed a significant decrease during the period 2001–2010, with an annual percent change (APC) of -5.5% (95%CI -8.3; -2.6). In turn, the average annual percent change (AAPC) of -5.1% (95%CI -10.5; 0.6) indicated stability over the entire period. Similar stability trends were observed in the incidence rates of lymphomas (AAPC -2.4%; 95%CI -5.0; 0.3), central nervous system tumors (AAPC 0.8%; 95%CI -3.2; 4.9), and other tumors. Only cases of soft tissue sarcomas showed a significant decrease in the average annual percent change (AAPC -7.3%; 95%CI -10.6; -3.8) during the period 2001–2018. The trends in age-standardized mortality rates for all diagnostic groups remained stable (Table 2). 

**Table 2:** Trend and annual percent change (APC), average annual percent change (AAPC), and 95% confidence intervals (95%CI) of incidence and mortality rates of the five most common types of cancer among children and adolescents. Mato Grosso, 2001–2018 (n=2,771)

Cause	n (%)	Period	APC (95%CI)	AAPC (95%CI)
Incidence				
Leukemia	579 (30.23)	2001–2010	-5.5 (-8.3; -2.6)	-5.1 (-10.5; 0.6)
		2010–2015	6.5 (-4.6; 19.0)	
		2015–2018	-20.8 (-42.8; 8.7)	
Lymphomas	268 (13.99)	2001–2018	-2.4 (-5.0; 0.3)	-2.4 (-5.0; 0.3)
Central nervous system tumors	305 (15.93)	2001–2018	0.8 (-3.2; 4.9)	0.8 (-3.2; 4.9)
Soft tissue sarcomas	101 (5.27)	2001–2018	-7.3 (-10.6; -3.8)	-7.3 (-10.6; 3.8)
Bone tumors	151 (7.89)	2001–2005	-10.4 (-34.9; 23.2)	-3.1 (-10.5; 5.0)
		2005–2018	-0.7 (-6.4; 5.4)	
Mato Grosso	1,915 (100.0)	2001–2016	-1.1 (-2.0; -0.2)	-3.1 (-8.6; 2.8)
		2016–2018	-16.9 (-51.8; 43.4)	
Mortality				
Leukemia	278 (34.92)	2001–2018	-0.4 (-2.8; 2.0)	-0.4 (-2.8; 2.0)
Lymphomas	68 (8.54)	2001–2018	0.1 (-3.6; 4.0)	0.1 (-3.6; 4.0)
Central nervous system tumors	181 (22.74)	2001–2018	-0.4 (-4.0; 5.0)	-0.4 (-4.0; 5.0)
Soft tissue sarcomas	42 (5.28)	-	-	-
Bone tumors	57 (7.16)	2001–2018	-0.1 (-4.1; 4.0)	-0.1 (-4.1; 4.0)
Mato Grosso	796 (100.0)	2001–2018	-0.8 (-2.6; 1.0)	-0.8 (-2.6; 1.0)

## Discussion

This study revealed a decreasing trend in age-standardized incidence rates for leukemias and soft tissue sarcomas, and stability for the other cancer types between 2001 and 2018. The age-standardized mortality rates remained stable for all analyzed cancer types. Both new cases and deaths were higher among male adolescents aged 15–19 years, and of Brown race/skin color. The most frequent diagnostic groups were, respectively, leukemias, lymphomas, central nervous system tumors, soft tissue sarcomas, and bone tumors.

Two limitations must be considered when interpreting the results. The first limitation concerns the use of secondary data to estimate incidence and mortality, as such data may be subject to information bias resulting from underreporting or recording errors. The other limitation refers to the impossibility of generalizing the findings to other states and regions of Brazil.

Despite the reported limitations, the results of this study are consistent with the literature. The types of cancer most frequently observed in this study were also reported in high-, middle-, and low-income countries (18–20). These types of cancer were leukemias, lymphomas, central nervous system tumors, soft tissue sarcomas, and bone tumors.

This study found a decrease in incidence rates for leukemias and stability for the other most common types of cancer in Mato Grosso. Even with the decrease or stability, the rates found were higher than those reported for Latin America and the Caribbean for central nervous system neoplasms (23.0 per 1,000,000 inhabitants) and lymphomas (16.6 per 1,000,000 inhabitants) (21). In Goiás, stability in incidence rates was also observed between 1996 and 2012 (AAPC -0.5; 95%CI -2.4; 1.4) (22). 

Evidence indicated an increase in cancer incidence rates in high-income countries, among children for leukemia, non-Hodgkin lymphomas, and central nervous system tumors (20,21), and adolescents for leukemia, central nervous system tumors, and epithelial carcinomas (23). The literature has reported that epidemiological, clinical profile, and data differences between high-, middle-, and low-income countries may result from regional socioeconomic disparities and the level of delivery of health care (1,10,24,25). 

Between 1996 and 2017, in the Brazilian Central-West region, cancers were the second leading cause of death among children and adolescents aged 0–19 years and accounted for 8,942 deaths among those aged 0–4 years (10). These findings revealed a trend of stability for the analyzed period regarding the mortality rate. A similar result was found in Goiânia, where stability was observed (AAPC 0.0; 95%CI -2.6; 2.7) for the same indicator (22). It is noteworthy that Mato Grosso and Goiás are located in the same Brazilian region, showing similarities in their agro-export-oriented economy and the health care network for cancer patients, which may partially explain the convergence in results for both incidence and mortality (10).

A study that analyzed cancer mortality rates among children and adolescents between 1996 and 2017 in the 133 intermediate Brazilian regions identified increasing trends in the North and Northeast regions (26). Regional differences in cancer mortality among Brazilian children and adolescents may reflect inequalities in access to diagnosis. This is due to the limited number of specialized services distributed across the country, creating gaps in health care provision that hinder the possibility of timely treatment initiation and, consequently, contribute to the reduction of mortality rates (27).

The sociodemographic profile with the highest frequency of deaths was male sex in the 15–19-year age group. This scenario differs from evidence that identified a higher proportion of deaths in the 0–4-year age group in different Brazilian regions (26). These findings are consistent with the profiles reported by Brazilian studies included in literature reviews (28). 

The stability in incidence and mortality rates observed in this study may result from different factors, such as the rarity of neoplasms in this age group combined with underdiagnosis of cases. It is estimated that 43.0% of cases are underdiagnosed worldwide (29). In addition to underdiagnosis, case monitoring is hindered by the quality of the available data. Disparities in the quality of cancer data are evident among the health regions of Mato Grosso, highlighting the need for continuous improvement in the quality of information from the state’s population-based cancer registries. This situation undermines improvement actions, the allocation of financial resources to hospital units that provide diagnosis and treatment in the coverage area, and cancer registration in the state (30).

The low number of annual cases may lead to fluctuations in rates and the appearance of random patterns, making it difficult to understand cancer trends among children and adolescents. This pattern may result, on one hand, from the registration of new cases attended and recorded exclusively in public health services or treated outside the municipality of residence; and, on the other hand, from early diagnosis of the disease, increased survival of children and adolescents with the disease, or improved adherence to appropriate treatment (23,27,30). 

Among the strengths of this study, the results may contribute to a better understanding of etiology and the development of strategies for prevention and early diagnosis. The study advanced the analysis of cancer trends among children and adolescents over 17 years in a state whose agro-export-oriented, chemical-dependent economic characteristics may contribute to increased environmental exposure to carcinogenic products and, consequently, to disease risk (8,9). This finding may also reveal similar patterns that can be observed in locations with the same economic base, as evidenced in Goiânia (22). Although this study did not analyze the association between pesticide exposure and the occurrence of cancer in the 0–19 age group, the literature has presented robust evidence of this relationship (9–10). Finally, this finding contributes to the monitoring of childhood and adolescent cancers in Mato Grosso, helping to address the challenges the state has faced in implementing effective cancer surveillance (30). 

In Mato Grosso, there are only two hospital units considered high-complexity oncology care centers authorized for pediatric oncology services—one of them is located in the state capital, Cuiabá (27). Accurate diagnosis and the initiation of appropriate treatment constitute significant challenges in the effort to improve survival rates in this age group (22,30). In this regard, it is essential to strengthen health systems to enhance early detection of cases, promote timely access to treatment, and ensure the registration of new cases (18). 

These findings showed stability in incidence and mortality rates, which should not be interpreted as an improvement in the temporal trend of cancers among children and adolescents in Mato Grosso. Despite the seemingly positive results, the rates found are excessively high when compared to other countries in Latin America and the Caribbean. In this context, there is a clear need for awareness campaigns and preventive actions focused on early detection, expansion of specialized service networks, and strengthening primary care through training aimed at improving data quality, early diagnosis, and appropriate treatment for this population.

The importance of further studies investigating factors associated with childhood and adolescent cancer across all Brazilian regions is emphasized, as well as the quality of information available in publicly accessible databases. This will contribute to a better understanding of the stability in mortality and incidence rates demonstrated in this study.
