# Supplementary material for: Trend in cancer incidence and mortality among children and adolescents in Mato Grosso, 2001–2018
Source: Epidemiol Serv Saude. 2025 Oct 3;34:e20250146. doi: 10.1590/S2237-96222025v34e20250146.en (PMC12494372; doi:10.1590/S2237-96222025v34e20250146.en)
Supplement: Translation

# Translation: Tendência da incidência e mortalidade por câncer entre crianças e adolescentes em Mato Grosso, 2001-2018

**DOI:** 10.1590/S2237-96222025v34e20250146.pt

**Authors:** Mariana Rosa Soares, Pablo Cardozo Roccon, Wanderlei Antonio Pignati, Noemi Dreyer Galvão, Paulo César Fernandes de Souza, Amanda Cristina de Souza Andrade

**Affiliations:** 1 Universidade Federal de Mato Grosso, Instituto de Saúde Coletiva, Cuiabá, MT, Brasil; 2 Secretaria de Saúde do Estado de Mato Grosso, Cuiabá, MT, Brasil; 3 Instituto René Rachou, Fundação Oswaldo Cruz, Belo Horizonte, MG, Brasil

**Keywords:** Incidência, Câncer, Criança, Adolescente, Estudos de Séries Temporais

## Abstract

**Objetivo:**

Analisar a tendência temporal da incidência e mortalidade por câncer entre crianças e adolescentes residentes em Mato Grosso no período 2001-2018.

**Métodos:**

Tratou-se de estudo de séries temporais das taxas de incidência e mortalidade por câncer entre crianças e adolescentes de 0-19 anos residentes em Mato Grosso. Para as estimativas de variações percentuais anuais (APC) e intervalos de confiança de 95% (IC95%), foi utilizada a regressão segmentada.

**Resultados:**

Foram registrados 1.915 casos novos e 796 óbitos, sendo os mais incidentes as leucemias (30,23% dos casos e 34,92% dos óbitos) na faixa etária de 15-19 anos (28,25% dos casos e 30,78% dos óbitos), na raça/cor da pele parda (45,17% dos casos e 50,75% dos óbitos) e no sexo masculino (53,89% dos casos e 56,53% dos óbitos). As taxas de incidência apresentaram tendência decrescente para as leucemias entre 2001 e 2010 (APC -5,5%; IC95% -8,3; -2,6) e para os sarcomas de partes moles (APC-7,3%; IC95% -10,6; -3,8) no período 2001-2018, assim como para todas as causas entre 2001 e 2016 (APC -1,1%; IC95% -2,0; -0,2). As taxas de mortalidade mantiveram estabilidade em todo o período e para todos os tipos de câncer.

**Conclusões:**

Apesar de apresentar estabilidade, as taxas em Mato Grosso são altas, o que requer estratégias de promoção do acesso ao diagnóstico precoce e ao tratamento adequado em tempo oportuno, como a ampliação da rede de serviços especializados, a melhoria na qualidade desses serviços e a atualização dos dados.

**Aspectos éticos**

A pesquisa respeitou os princípios éticos, obtendo os seguintes dados de aprovação:

Comitê de ética em pesquisa: Universidade Federal de Mato Grosso

Número do parecer: 4.858.521

Data de aprovação: 20/7/2021

Certificado de apresentação de apreciação ética: 48121421.0.0000.8124

Registro do consentimento livre e esclarecido: Dispensado.

## Introdução

O câncer infantojuvenil é uma doença crônica que acomete crianças e adolescentes na faixa etária de 0-19 anos (1). Nessa faixa etária, os tipos de tumores mais incidentes são leucemias, linfomas, tumores de sistema nervoso central, sarcomas e tumores ósseos (2).

No cenário global, percebe-se o aumento na incidência e mortalidade por câncer entre crianças e adolescentes, com ocorrência de mais de 400 mil casos novos e 73 mil mortes. Desse total, 80,0% ocorrem em países com baixo índice de desenvolvimento humano (3). No Brasil, estimam-se 4.230 casos para o sexo masculino e 3,7 mil para o sexo feminino para o triênio 2023-2025 (1). Para esse mesmo período, as maiores taxas brutas de incidência foram estimadas no Sul, com 152,26/1 milhão de habitantes, seguido do Sudeste (144,88/1 milhão de habitantes) e do Centro-Oeste (136,21/1 milhão de habitantes), onde localiza-se Mato Grosso, estado que possui a taxa bruta de 115,41/1 milhão de habitantes (1). Em Mato Grosso, entre os tipos mais incidentes no período 2001-2017, destacaram-se as leucemias e os linfomas, com taxas de incidência ajustada de 29,8 e 13,9 casos/1 milhão de habitantes (4).

Diferentemente dos cânceres em adultos, nessa faixa etária, os tumores possuem características clínicas próprias, em razão de sua origem etiológica, comportamento clínico e fatores associados (5). No que condiz ao comportamento clínico e prognóstico, observa-se a melhora nos índices de cura e redução de óbitos em países desenvolvidos. Isso decorre do melhor acesso ao diagnóstico precoce – a partir de sinais e sintomas que inicialmente podem ser muito inespecíficos – e do tratamento oportuno, os quais corroboram para a estabilidade das taxas de mortalidade nessas localidades (1,4,5). Em relação aos fatores associados, destacam-se as predisposições genética, hereditária e imunológica, a exposição ambiental a agentes genotóxicos, as radiações ionizantes, os campos eletromagnéticos, o tabaco, o alcoolismo na fase pré-concepção (6,9) e a exposição parental a agentes cancerígenos nos ambientes domiciliar e ocupacional (7-9).

Considerando os efeitos das exposições ambiental e ocupacional parental a agentes genotóxicos na ocorrência do câncer entre crianças e adolescentes (6-9), analisar o estado de Mato Grosso é de grande relevância científica e sanitária. Mato Grosso é um importante produtor brasileiro de commodities agrícolas sob caráter químico-dependente da cadeia produtiva do agronegócio e consome anualmente grandes toneladas de agrotóxico (9,10). 

A literatura tem evidenciado casos de cânceres em adultos e crianças decorrentes da exposição ambiental por residir à distância de 90 a 300 metros de lavouras em municípios de grande produção agrícola nesse estado e da exposição ocupacional parental nas fases de preconcepção ou gestação (9). Nesse sentido, crianças e adolescentes que residem em áreas de cultivo ou proximidades podem estar expostos aos efeitos genotóxicos dos agrotóxicos pela exposição causada pela deriva e pelo contato com água e solo contaminados (10). 

Este artigo teve como objetivo analisar a tendência temporal da incidência e mortalidade dos cinco tipos de câncer mais incidentes entre crianças e adolescentes residentes em Mato Grosso entre 2001 e 2018.

## Métodos

### Delineamento e contexto

Tratou-se de estudo de séries temporais com dados dos 141 municípios mato-grossenses. Mato Grosso está localizado na porção central do país e possui extensão territorial de 903.208,362 km^2^, população estimada de 3.658.649 habitantes e índice de desenvolvimento humano de 0,736, além de densidade demográfica de 4,05 habitantes/km^2^ (11).

### População e variáveis

O estudo foi composto por dados de crianças e adolescentes residentes e nascidos nos 141 municípios de Mato Grosso, com idade entre 0-19 anos que tiveram o diagnóstico de câncer, e por registros de óbitos por câncer na mesma faixa etária ocorridos entre 2001 e 2018. O recorte temporal decorreu em razão da disponibilidade e da atualização dos registros de câncer de base populacional do Instituto Nacional de Câncer.

As variáveis sociodemográficas incluíram: faixa etária em anos (<1, 0-4, 5-9, 10-14 e 15-19), sexo (feminino, masculino), raça/cor da pele (amarela, branca, indígena, parda, preta) e tipo de câncer segundo grupo de diagnóstico de acordo com International Classification of Childhood Cancer (12).

### Fontes de dados e mensuração

Os dados de incidência foram extraídos do Sistema de Registro de Câncer de Base Populacional, disponíveis na página eletrônica BasepopWeb (13). Os dados de óbito foram retirados do Sistema de Informações sobre Mortalidade (14), cedidos pela Secretaria de Estado de Saúde de Mato Grosso. As estimativas populacionais intercensitárias foram obtidas no Departamento de Informática do Sistema Único de Saúde, dados de domínio público (15). 

Foram calculadas as taxas de incidência e mortalidade (por 1 milhão de habitantes na faixa etária de 0-19 anos) para cada ano entre 2001 e 2018 e para os cinco grupos de diagnóstico mais incidentes. As taxas brutas específicas foram obtidas para cada faixa etária utilizando-se intervalos de cinco anos e padronizadas por idade, pelo método direto, considerando-se a população padrão mundial (16). 

### Métodos estatísticos

Para a análise de tendência, foi utilizada a regressão segmentada considerando o ano-calendário como variável regressora. Foram calculadas a variação percentual anual (*annual percent change* – APC) e a variação percentual média anual (*average annual percent change* – AAPC) – média geométrica ponderada das diferentes variações percentuais anuais com peso igual ao tamanho do segmento para cada intervalo de tempo (17). Foram estimados os respectivos intervalos de confiança de 95% (IC95%). Por ter apresentado valores nulos, a regressão segmentada para a taxa de mortalidade por sarcomas de partes moles não foi realizada. Foi considerado o nível de significância de 5%.

As taxas e os gráficos foram calculados e produzidos com o auxílio do software estatístico R versão 3.3.0. As análises foram realizadas com o software Joinpoint Regression Program, versão 8.3.6.1 (Statistical Research and Applications Branch, National Cancer Institute, Bethesda, Estados Unidos).

## Resultados

Entre 2001 e 2018, ocorreram 1.915 casos novos e 796 óbitos por câncer infantojuvenil em Mato Grosso. As maiores proporções foram observadas na faixa etária de 15-19 anos (28,25% dos casos e 30,78% dos óbitos) e no sexo masculino (53,89% dos casos e 56,53% dos óbitos). A raça/cor da pele parda representou 45,17% de casos incidentes e 50,75% dos óbitos. Os casos mais incidentes de câncer foram leucemias (30,23%), tumores do sistema nervoso central (15,93%), linfomas (13,99%), tumores ósseos (7,89%) e sarcomas de partes moles (5,27%). Entre os tipos de câncer dos óbitos, destacaram-se leucemias (34,92%), tumores do sistema nervoso central (22,74%), linfomas (8,54%), tumores ósseos malignos (7,89%) e sarcomas de tecidos moles e outros sarcomas extraósseos (5,28%) (Tabela 1).

**Tabela 1:** Frequências absoluta e relativa dos casos novos e óbitos por câncer entre crianças e adolescentes. Mato Grosso, 2001-2018 (n=2.771)

Variáveis	Casos novos	Óbitos
	n (%)	n (%)
**Faixa etária** (anos)		
<1	92 (4,80)	29 (3,64)
1-4	480 (25,07)	189 (23,74)
5-9	403 (21,04)	161 (20,23)
10-14	399 (20,84)	172 (21,61)
15-19	541 (28,25)	245 (30,78)
Sexo		
Feminino	883 (46,11)	346 (43,47)
Masculino	1.032 (53,89)	450 (56,53)
**Raça/cor da pele**		
Amarela	27 (1,41)	2 (0,25)
Branca	616 (32,17)	326 (40,95)
Indígena	21 (1,10)	13 (1,63)
Negra	78 (4,07)	28 (3,52)
Parda	865 (45,17)	404 (50,75)
Sem informação	308 (16,08)	23 (2,89)
Causa		
Leucemias	579 (30,23)	278 (34,92)
Linfomas e neoplasias reticuloendoteliais	268 (13,99)	68 (8,54)
Tumores do sistema nervoso central e neoplasias intracranianas e intraespinhais	305 (15,93)	181 (22,74)
Retinoblastoma	25 (1,31)	6 (0,75)
Tumores renais	99 (5,17)	14 (1,76)
Tumores hepáticos	20 (1,04)	14 (1,76)
Tumores ósseos malignos	151 (7,89)	57 (7,16)
Sarcomas de tecidos moles e outros sarcomas extraósseos	101 (5,27)	42 (5,28)
Tumores de células germinativas, tumores trofoblásticos e neoplasias de gônadas	92 (4,80)	20 (2,51)
Outras neoplasias epiteliais malignas e melanomas malignos	90 (4,70)	31 (3,89)
Outras neoplasias malignas não especificadas	103 (5,38)	35 (4,40)
Tumores do sistema nervoso simpático	82 (4,28)	50 (6,28)

As maiores incidência e mortalidade foram constatadas entre crianças com 0-9 anos quando comparadas aos juvenis com 10-19 anos. Para os grupos diagnósticos, verificaram-se incidência e mortalidade de 37,0% para leucemia e, nos casos de tumores do sistema nervoso central, incidência de 17,3% e mortalidade de 26,8% para a faixa etária de 0-9 anos. Na faixa etária de 10-19 anos, as maiores proporções de casos novos e óbitos concentraram-se em linfomas (16,5% e 9,4%) e tumores ósseos malignos (13,6% e 10,6%) (Figura 1).

**Figura 1:** Proporção de casos novos (A) e óbitos (B) por câncer entre crianças e adolescentes. Mato Grosso, 2001-2018 (n=2.771)

Observaram-se as taxas padronizadas de incidência para os cinco tipos de câncer mais frequentes (Figura 2). Para leucemias, a taxa de incidência foi 40,4 casos/1 milhão de habitantes em 2001, enquanto a de mortalidade foi 21,3/1 milhão de habitantes em 2003. No caso de linfomas, a incidência mais alta foi registrada em 2002, com 21,4 casos/1 milhão de habitantes, e a mortalidade mais elevada ocorreu em 2016, com 16,4/1 milhão de habitantes. Entre tumores do sistema nervoso central, a incidência foi maior em 2013, com 28,9 casos/1 milhão de habitantes, e a mortalidade também foi mais alta em 2013, com 19,2/1 milhão de habitantes. Sarcomas de tecidos moles apresentaram incidência de 6,4/1 milhão de habitantes e mortalidade de 4,9/1 milhão de habitantes em 2006. A maior incidência de tumores ósseos foi em 2008, com 11,5 casos/1 milhão de habitantes, e a mortalidade foi 6,5/1 milhão de habitantes em 2003.

**Figura 2:** Taxas de incidência (A) e mortalidade (B) padronizadas por idade dos cinco tipos de câncer mais incidentes (por 1 milhão de crianças e adolescentes). Mato Grosso, 2001-2018

A tendência da taxa padronizada de incidência de leucemias apresentou redução significativa no período 2001-2010, com variação percentual anual (APC -5,5% IC95% -8,3; -2,6). Por sua vez, a variação percentual média anual (AAPC -5,1%; IC95% -10,5; 0,6) indicou estabilidade para o período completo. Tendências de estabilidade similares foram observadas nas taxas de incidências de linfomas (AAPC -2,4%; IC95% -5,0; 0,3), tumores do sistema nervoso central (AAPC 0,8%; IC95% -3,2; 4,9) e outros tumores. Somente os casos de sarcomas de partes moles apresentaram redução significativa na variação percentual média anual (AAPC -7,3%; IC95% -10,6; -3,8) no período 2001-2018. As tendências das taxas padronizadas de mortalidade para todos os grupos de diagnósticos permaneceram estáveis (Tabela 2). 

**Tabela 2:** Tendência e variação percentual anual (APC), variação percentual média anual (AAPC) e intervalos de confiança de 95% (IC95%) da taxa de incidência e mortalidade dos cinco tipos mais incidentes de câncer entre crianças e adolescentes. Mato Grosso, 2001-2018 (n=2.771)

Causa	n (%)	Período	APC (IC95%)	AAPC (IC95%)
Incidência				
Leucemia	579 (30,23)	2001-2010	-5,5 (-8,3; -2,6)	-5,1 (-10,5; 0,6)
		2010-2015	6,5 (-4,6; 19,0)	
		2015-2018	-20,8 (-42,8; 8,7)	
Linfomas	268 (13,99)	2001-2018	-2,4 (-5,0; 0,3)	-2,4 (-5,0; 0,3)
Tumores do sistema nevoso central	305 (15,93)	2001-2018	0,8 (-3,2; 4,9)	0,8 (-3,2; 4,9)
Sarcomas de partes moles	101 (5,27)	2001-2018	-7,3 (-10,6; -3,8)	-7,3 (-10,6; 3,8)
Tumores ósseos	151 (7,89)	2001-2005	-10,4 (-34,9; 23,2)	-3,1 (-10,5; 5,0)
		2005-2018	-0,7 (-6,4; 5,4)	
Mato Grosso	1.915 (100,0)	2001-2016	-1,1 (-2,0; -0,2)	-3,1 (-8,6; 2,8)
		2016-2018	-16,9 (-51,8; 43,4)	
Mortalidade				
Leucemia	278 (34,92)	2001-2018	-0,4 (-2,8; 2,0)	-0,4 (-2,8; 2,0)
Linfomas	68 (8,54)	2001-2018	0,1 (-3,6; 4,0)	0,1 (-3,6; 4,0)
Tumores do sistema nervoso central	181 (22,74)	2001-2018	-0,4 (-4,0; 5,0)	-0,4 (-4,0; 5,0)
Sarcomas de partes moles	42 (5,28)	-	-	-
Tumores ósseos	57 (7,16)	2001-2018	-0,1 (-4,1; 4,0)	-0,1 (-4,1; 4,0)
Mato Grosso	796 (100,0)	2001-2018	-0,8 (-2,6; 1,0)	-0,8 (-2,6; 1,0)

## Discussão

Este estudo evidenciou a tendência decrescente nas taxas padronizadas de incidência de leucemias e sarcomas de partes moles e a estabilidade para os demais tipos de câncer entre 2001 e 2018. As taxas padronizadas de mortalidade apresentaram estabilidade para todos os tipos de câncer analisados. Tanto os casos novos quanto os óbitos se apresentaram maiores entre adolescentes com idade entre 15-19 anos, do sexo masculino e da raça/cor da pele parda. Os grupos diagnósticos mais frequentes foram, respectivamente, das leucemias, dos linfomas, dos tumores do sistema nervoso central, dos sarcomas de partes moles e dos tumores ósseos.

Duas limitações devem ser consideradas na interpretação dos resultados. A primeira delas é com relação à utilização de dados secundários para estimativas de incidência e mortalidade, visto que tais dados podem sofrer viés de informação decorrente de sub-registro, subnotificação ou erros no registro. A outra limitação é referente à impossibilidade de generalização dos achados para outros estados e regiões do Brasil.

Apesar das limitações relatadas, os resultados deste estudo ecoam na literatura. Os tipos de câncer com maior frequência evidenciados neste estudo também foram observados em países de alta, média e baixa rendas (18-20). Esses tipos de câncer foram leucemias, linfomas, tumores de sistema nervoso central, sarcomas de partes moles e tumores ósseos.

Este estudo constatou redução nas taxas de incidência para leucemias e estabilidade para os demais tipos de câncer mais incidentes em Mato Grosso. Mesmo diante da redução ou estabilidade, as taxas encontradas se apresentaram maiores que as taxas evidenciadas para a América Latina e Caribe quanto a neoplasias do sistema nervoso central (23,0/1 milhão de habitantes) e linfomas (16,6/1 milhão de habitantes) (21). Em Goiás, também foi evidenciada estabilidade, entre 1996 e 2012, nas taxas de incidência (AAPC -0,5; IC95% -2,4; 1,4) (22). 

Evidências apontaram aumento nas taxas de incidência por câncer, em países de alta renda, entre crianças para leucemia, linfomas não Hodgkin e tumores do sistema nevoso central (20,21) e, entre adolescentes, leucemias, tumores de sistema nervoso central e carcinomas epiteliais (23). A literatura tem reportado que as diferenças epidemiológicas, de perfil clínico e de dados entre países de alta, média e baixa rendas podem ser resultado das diferenças regionais socioeconômicas e do nível de assistência à saúde (1,10,24,25). 

Entre 1996 e 2017 no Centro-Oeste brasileiro, os cânceres representaram a segunda maior causa de mortalidade entre crianças e adolescentes na faixa etária de 0-19 anos e foram responsáveis por 8.942 óbitos entre aqueles com 0-4 anos (10). Estes achados revelaram tendência de estabilidade para o período analisado em relação à taxa de mortalidade. Resultado similar foi encontrado em Goiânia, onde observaram estabilidade (AAPC 0,0; IC95% -2,6; 2,7) para o mesmo indicador (22). Destaca-se que Mato Grosso e Goiás ocupam a mesma região brasileira, apresentando similaridades quanto à matriz econômica agroexportadora e à rede de atendimento a pacientes com câncer, o que pode parcialmente explicar a aproximação nos resultados tanto para a incidência quanto para a mortalidade (10).

Estudo que analisou as taxas de mortalidade por câncer entre crianças e adolescentes de 1996 e 2017 nas 133 regiões intermediárias brasileiras identificou tendências crescentes nas regiões Norte e Nordeste (26). As diferenças regionais na mortalidade por câncer em crianças e adolescentes brasileiros podem ser reflexo de desigualdades no acesso ao diagnóstico. Isso é decorrente de poucos serviços especializados distribuídos no país e forma bloco de vazios assistenciais que dificultam a possibilidade de iniciar o tratamento oportuno e, assim, contribuir para a redução das taxas de mortalidade (27).

O perfil sociodemográfico no qual foi encontrada a maior frequência de óbitos foi ser do sexo masculino na faixa etária de 15-19 anos. Esse cenário diverge das evidências que identificaram maior proporção de óbitos na faixa etária de 0-4 anos em diferentes regiões brasileiras (26). Estes achados convergem com os perfis reportados por estudos brasileiros incluídos em revisões de literatura (28). 

A estabilidade nas taxas de incidência e mortalidade observada neste estudo pode ser resultado de diferentes fatores, como a raridade das neoplasias nessa faixa etária somada ao subdiagnóstico dos casos. Estima-se que 43,0% dos casos são subdiagnosticados em todo o mundo (29). Além do subdiagnóstico, os monitoramentos dos casos são prejudicados pela qualidade dos dados disponíveis. Evidenciam-se disparidades na qualidade dos dados sobre câncer entre as regiões de saúde de Mato Grosso, o que indica a necessidade de melhoria contínua da qualidade das informações dos registros de câncer de base populacional do estado. Essa realidade compromete as ações de aprimoramento, o repasse de recursos financeiros para as unidades hospitalares que realizam diagnóstico e tratamento na área de cobertura e o registro do câncer no estado (30).

O número reduzido de casos anuais pode levar a flutuações nas taxas e na apresentação de padrões aleatórios, o que dificulta a compreensão das tendências do câncer entre crianças e adolescentes. Esse padrão pode resultar, por um lado, do padrão de registros dos casos novos atendidos e registrados exclusivamente nos serviços públicos de saúde ou tratados fora do município de residência; por outro, do diagnóstico precoce da doença e do aumento da sobrevida das crianças e adolescentes com o diagnóstico da doença, ou na melhora na adesão tratamento adequado (23,27,30). 

Das potencialidades deste estudo, os resultados podem contribuir para melhor compreensão da etiologia e elaboração de estratégias de prevenção e diagnóstico precoce. O estudo promoveu avanço na análise da tendência do câncer entre crianças e adolescentes no período de 17 anos de um estado cujas características econômicas agroexportadora químico-dependente podem contribuir para o aumento à exposição ambiental a produtos cancerígenos e, assim, ao risco de doença (8,9). Este achado também pode revelar padrões similares passíveis de serem encontrados em locais com a mesma base econômica, como evidenciado em Goiânia (22). Ainda que neste estudo não tenha sido analisada a associação entre as exposições a agrotóxicos e a ocorrência de câncer na faixa etária de 0-19 anos, a literatura tem apresentado evidências robustas dessa relação (9-10). Por fim, este achado contribui para o monitoramento dos cânceres infantojuvenis em Mato Grosso, auxiliando na minimização dos problemas que esse estado tem enfrentado para a efetivação da vigilância do câncer (30). 

Em Mato Grosso, existem apenas duas unidades hospitalares que são consideradas unidades de assistência de alta complexidade em oncologia habilitadas para o serviço de oncologia pediátrica – uma delas se localiza na capital do estado, Cuiabá (27). O diagnóstico correto e o início do tratamento adequado constituem-se desafios significativos na busca por melhorar as taxas de sobrevida nessa faixa etária (22,30). Nesse sentido, é primordial fortalecer os sistemas de saúde para ampliar a detecção precoce dos casos e promover o acesso tempestivo ao tratamento e o registro dos casos novos (18). 

Estes achados demonstraram estabilidade nas taxas de incidência e óbito que não devem ser interpretadas como melhora na tendência temporal dos cânceres entre crianças e adolescentes em Mato Grosso. Apesar dos resultados aparentemente positivos, as taxas encontradas se revelam demasiadamente altas quando comparadas a outros países da América Latina e Caribe. Nesse sentido, destacam-se a necessidade de ações e campanhas de conscientização sobre prevenção e detecção precoce dos casos, a ampliação da rede de serviços especializados e a capacitação da Atenção Primária em busca da melhoria da qualidade dos dados, do diagnóstico precoce e do tratamento adequado para essa população.

Reforça-se a importância de novos estudos que investiguem fatores associados ao câncer infantojuvenil em todas as regiões brasileiras, bem como a qualidade da informação disponível em bases de domínio público. Isso contribuirá para a maior compreensão da estabilidade nas taxas de mortalidade e incidência evidenciadas neste estudo.
